# Prevalence of Vector-Borne Pathogens in Reproductive and Non-Reproductive Tissue Samples from Free-Roaming Domestic Cats in the South Atlantic USA

**DOI:** 10.3390/pathogens10091221

**Published:** 2021-09-21

**Authors:** Charlotte Manvell, Kelli Ferris, Ricardo Maggi, Edward B. Breitschwerdt, Erin Lashnits

**Affiliations:** 1 Intracellular Pathogens Research Laboratory, Comparative Medicine Institute, College of Veterinary Medicine, North Carolina State University, Raleigh, NC 27606, USA; comanvel@ncsu.edu (C.M.); rgmaggi@ncsu.edu (R.M.); ebbreits@ncsu.edu (E.B.B.); 2 Department of Clinical Sciences, College of Veterinary Medicine, North Carolina State University, Raleigh, NC 27606, USA; kkferris@ncsu.edu; 3 Department of Medical Sciences, School of Veterinary Medicine, University of Wisconsin, Madison, WI 53706, USA

**Keywords:** *Bartonella* spp., hemotropic *Mycoplasma* spp., reproduction, cats, prevalence

## Abstract

Reservoir to multiple species of zoonotic pathogens, free-roaming cats (FRCs) interact with domestic and wild animals, vectors, and humans. To assess the potential for feline vector-borne pathogens to be vertically transmitted, this study surveyed ear tip and reproductive tissues of FRCs from two locations in the South Atlantic United States for *Anaplasma, Bartonella*, *Ehrlichia,* hemotropic *Mycoplasma*, and *Rickettsia* species. We collected ovary (*n* = 72), uterus (*n* = 54), testicle (*n* = 74), and ear tip (*n* = 73) tissue from 73 cats, and fetal (*n* = 20) and placental (*n* = 19) tissue from 11 queens. Pathogen DNA was amplified utilizing qPCR, confirmed by sequencing. Cats were more frequently *Bartonella henselae* positive on reproductive tissues (19%, 14/73) than ear tip (5%, 4/73; *p* = 0.02). *B. henselae* was amplified from fetus (20%, 4/20) and placenta samples (11%, 2/19). *Bartonella* spp. infection was more common in cats from North Carolina (76%, 26/34) than Virginia (13%, 5/39; *p* < 0.0001). Fourteen percent (10/73) of both ear tip and reproductive tissues were positive for hemotropic *Mycoplasma* spp. *Anaplasma, Ehrlichia*, and *Rickettsia* spp. DNA was not amplified from any cat/tissue. These findings suggest that *B. henselae* preferentially infected cats’ reproductive tissue and reinforces the importance of investigating the potential for *B. henselae* vertical transmission or induction of reproductive failure.

## 1. Introduction 

Free roaming cats (FRC) exist in a unique position, regularly interacting with a diverse assortment of species including wild predators and prey, domestic animals and pets, arthropod vectors, and humans that often care for them. Each of these interfaces represents a unique opportunity for inter-species pathogen transmission. The cat’s status as a known or suspected reservoir host for multiple vector-borne pathogens [1,2,3], including *Bartonella* [4,5,6], hemotropic *Mycoplasma* [7], and *Rickettsia* [2] species increases the importance of understanding these diseases in FRC populations. 

Identified as emerging pathogens of both animals and humans, the genus *Bartonella* comprises 45 species or subspecies, 13 of which are known to cause human disease [8]. Of these species, *Bartonella henselae*, *Bartonella clarridgeiae*, and *Bartonella koehlerae* are known to employ the cat as a reservoir host [4,5,6]. Isolation of four other *Bartonella* spp. from cat blood and tissue has also been documented [9,10,11,12]. Clinical manifestations of *Bartonella* spp. infection in the cat host are uncommon; however, associations have been made with central nervous system disease, ocular disease, immune complex disease, and endomyocarditis on the basis of epidemiologic studies and case reports [13,14,15,16,17]. *B. henselae*, as well as potentially other *Bartonella* spp. associated with the cat, are primarily vectored by *Ctenocephalides felis*, the cat flea [18].

Multiple *Mycoplasma* spp. are found in cats and can be classified as either respiratory or hemotropic in nature. Given our vector-borne focus, we chose to evaluate only the hemotropic *Mycoplasma* spp., three of which are commonly found in cats: *Mycoplasma haemofelis* (Mhf), *Candidatus* Mycoplasma haemominutum (Mhm), and *Candidatus* Mycoplasma turicensis (Mtc) [19]. Disease manifestations caused by hemotropic *Mycoplasma* spp. in cats, including lethargy, inappetence, tachycardia, and tachypnea, are typically related to the induction of hemolytic anemia [19]. Though not widely regarded as zoonotic in humans, Mhf infection in an HIV-positive patient co-infected with *B. henselae* has been reported, suggesting that Mhf may have zoonotic potential [20]. Historically assumed to be a vector-borne, specifically a flea-borne disease, the mechanism of hemotropic *Mycoplasma* spp. transmission between cats is currently unknown; however, direct transmission due to fighting is now suspected due to the strong association between infection and male sex and lack of evidence of flea-borne transmission [7,21,22,23]. An experimental transmission study using *C. felis* to transmit hemotropic *Mycoplasma* spp. resulted in only one of six recipient cats becoming transiently infected with Mhf and none of three recipient cats becoming infected with Mhm [24]. 

Pertinent to the present study is the knowledge gap regarding the role of vertical transmission of many vector-borne pathogens in maintaining reservoirs of infection. Reports vary widely based on pathogen and host, with evidence lacking for vertical transmission in *Ehrlichia ewingii* and *Anaplasma phagocytophilum* in dogs [25,26] and *Cytauxzoon felis* [27] in cats and evidence in favor of vertical transmission of *Anaplasma platys, Babesia gibsoni, Borrelia burgdorferi, Leishmania infantum*, and *Trypanosoma cruzi* in dogs [28,29,30,31,32,33,34], *Babesia microti* in humans and voles [32,35,36], and *Hepatozoon canis* in red foxes and dogs [37,38]. The possibility of vertical transmission is of particular importance for *Bartonella* spp., which have been suggested as a cause of reproductive failure in multiple species. A study by Guptill et al. reported *B. henselae* as a cause of reproductive failure in the domestic cat [39]. Few other studies have investigated the relationship between *Bartonella* spp. and reproduction. Boulouis et al. reported *Bartonella birtlesii* infection in pregnant mice having an abortifacient effect as well as increasing the rate of fetal resorption and death and decreasing fetal weight gain [40]. A case report documenting *B. henselae* DNA amplification and sequencing from a mother’s blood and cervical tissue, as well as her neonatal daughter’s brain and liver samples acquired during autopsy suggested that *B. henselae* could play a role in reproductive failure and neonatal loss in humans [41]. Unraveling the relationship between *Bartonella* spp. and reproduction is critical to determine their potential to interfere with reproductive function in a variety of species as well as be transmitted vertically as a means of non-vector-borne infection and maintenance in the reservoir host. 

The primary objective of this study was to determine the prevalence of feline vector-borne pathogens, including *Bartonella*, hemotropic *Mycoplasma*, and *Rickettsia* spp., as well as *Anaplasma* and *Ehrlichia* spp. in the reproductive and ear tip tissues of FRCs in two distinct geographic locations. Due to previous literature suggesting *Bartonella* spp. as a cause of reproductive failure, we expected *Bartonella* spp. to be more common in the reproductive tissues than the ear tip tissues, while the proportion of samples with DNA from other feline vector-borne diseases was not expected to vary between ear tip and reproductive tissues. We also expected pathogen prevalence in cats to be consistent between the two geographic locations. The secondary objective was to determine if *Bartonella* spp. DNA was present in fetal cat tissues. 

## 2. Results

### 2.1. Feline VBDs Detected 

**Figure 1:**
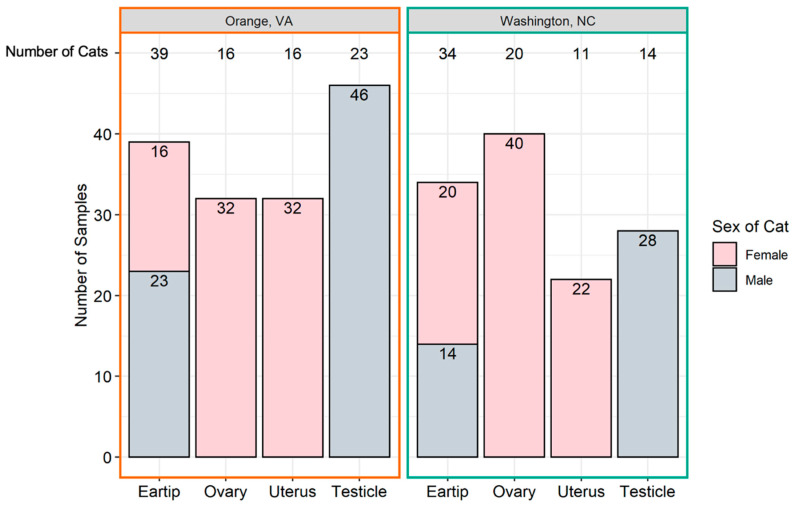
Number of tissue samples collected. The total number of samples (y-axis) originating from each tissue (x-axis) is represented above by geographic location. The total number of cats from which each sample type was collected is at the top of the graph.

Ear tip and reproductive tissues were obtained from 73 cats, including 36 females (49%) and 37 males (51%). The number and type of samples obtained from each location is provided in Figure 1. Thirty-nine cats were from Orange, VA (54%) and 34 were from Washington, NC (46%). The proportion of female and male cats from Orange, VA and Washington, NC were not significantly different (*p* = 0.16). The mean weight of all cats was 3.13 kg; Orange, VA cats had a mean weight of 3.21 kg (standard deviation 1.13 kg), whereas Washington, NC cats had a mean weight of 3.04 kg (standard deviation 0.9 kg); there was no significant difference in weight by location (*p* = 0.44). Male cats (mean 3.46 kg, standard deviation 1.22 kg) were heavier than female cats (mean 2.73 kg, standard deviation 0.55 kg; *p* < 0.0001). Females had a mean number of 4.5 tissues tested (range 3–5), while all males had three tissues tested. 

Thirty-eight of 73 cats (52%) were qPCR positive for one or more pathogen on one or more tissue sample. Forty-two percent (31/73) of cats were positive for one or more *Bartonella* spp. by qPCR, and 16% (12/73) of cats were positive for one or more hemotropic *Mycoplasma* spp. by qPCR. *Anaplasma, Ehrlichia*, and *Rickettsia* spp. DNA was not amplified from any of the tissues tested from any cat.

### 2.2. Bartonella spp. 

**Figure 2:**
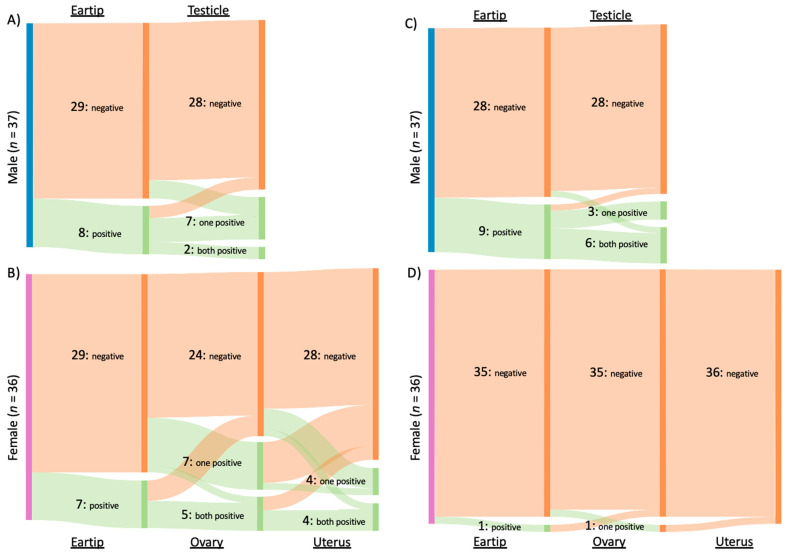
Number and type of tissue from which *Bartonella* spp. and Hemotropic *Mycoplasma* spp. were amplified. Type of tissue samples from which *Bartonella* spp. (**A**,**B**) or hemotropic *Mycoplasma* spp. (**C**,**D**) DNA was amplified from male (**A**,**C**) and female (**B**,**D**) cats. Each column represents a tissue (indicated at top or bottom) with the number cats of negative, one of two tissues positive, or both tissues positive indicated by the labelled bar. x.

Of the 31 cats infected with *Bartonella* spp., *B. henselae* was amplified from 16 cats, *B. clarridgeiae* from 15 cats, and *B. koehlerae* from two cats. Both *B. clarridgeiae* and *B. henselae* DNA were amplified from 10% (3/31) of infected cats, all of which were males from Washington, NC. When considering all *Bartonella* spp. together, 34% (25/73) of cats were qPCR positive from reproductive tissue, compared to 21% (15/73) that were qPCR positive from ear tip tissue (*p* = 0.09). The type of tissues that tested positive for *Bartonella* spp. infection is summarized in Figure 2A and 2B for male and female cats, respectively. 

**Figure 3:**
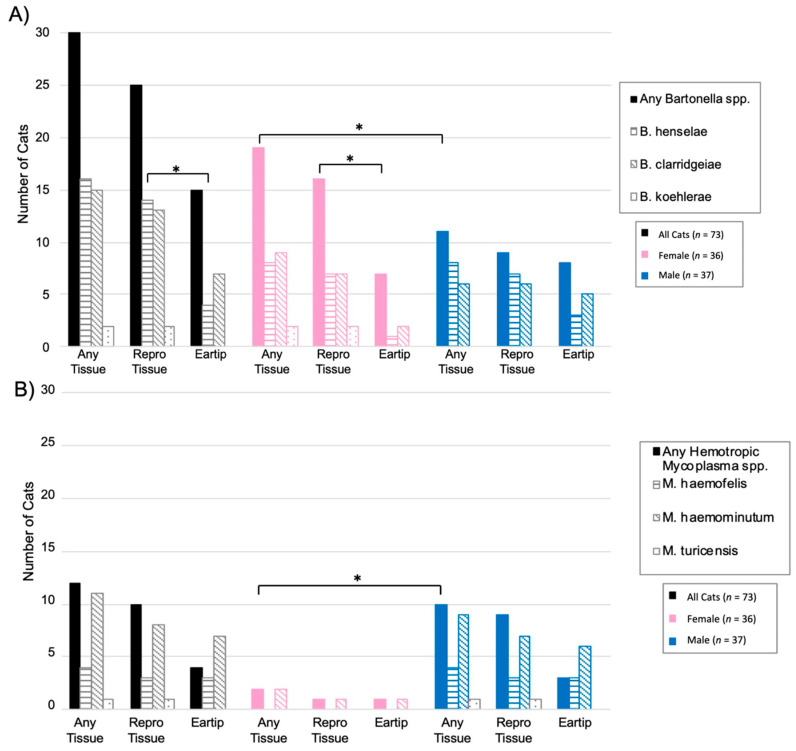
Number of cats infected with *Bartonella* spp. and hemotropic *Mycoplasma* spp. by tissue type. (**A**) Total number of cats positive for any *Bartonella* spp., *B. henselae*, *B. clarridgeiae*, and *B. koehlerae* on either ear tip or reproductive tissues by sex. (**B**) Total number of cats positive for any hemotropic *Mycoplasma* spp., Mhf, Mhm, and Mtc on either ear tip or reproductive tissues by sex. * Statistical significance considered at *p* < 0.05 by Fisher’s exact test.

When considering each *Bartonella* species separately, significantly more cats were positive on reproductive tissue (14/73, 19%) than ear tip (4/73, 5%) for *B. henselae* (*p* = 0.02). Of the four cats with *B. henselae*-positive ear tip tissues, reproductive tissue(s) were also positive for two cats. In comparison, *B. clarridgeiae* was found in similar proportions from reproductive tissues (9/73, 12%) and ear tip tissue (11/73, 15%; *p* = 0.81). *Bartonella koehlerae* was only detected in two cats, both female from Washington, NC, with DNA being amplified from either ovary or uterine tissue, but not ear tip tissue. Figure 3A summarizes *Bartonella* spp. qPCR results from each tissue type. *B. henselae* and *B. clarridgeiae* DNA was amplified from all included tissue types, while *B. koehlerae* DNA was only amplified from the ovary or uterus.

**Figure 4:**
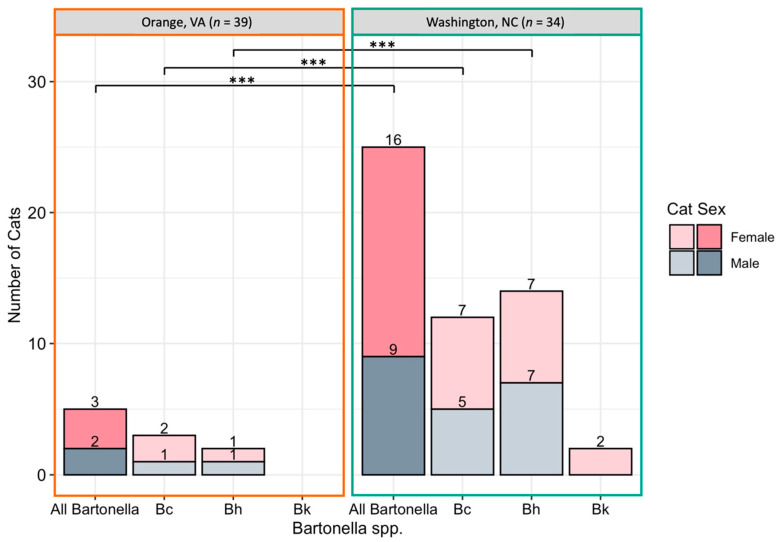
Number of cats infected with *Bartonella* spp. by location. Cats were considered positive if one or more tissue samples were qPCR positive for all *Bartonella* spp., *B. clarridgeiae* (Bc), *B. henselae* (Bh), or *B. koehlerae* (Bk). *** Statistical significance considered at *p* < 0.0001 by Fisher’s exact test.

*Bartonella* spp. DNA was more often amplified from female cats than male cats with 56% (20/36) of female cats *Bartonella* spp. qPCR positive by one or more tissue, compared to 30% (11/37) of male cats (*p* = 0.03; Figure 3A *Bartonella* infection was significantly more common in cats sampled at the North Carolina location than the Virginia location, with 78% (29/37) of cats from North Carolina *Bartonella* spp. qPCR positive compared to only 13% (5/39) of cats from Virginia (*p* < 0.0001). *Bartonella* spp. detection at each location is displayed in Figure 4. There was a significant difference in the prevalence of both *B. henselae* (*p* < 0.001) and *B. clarridgeiae* (*p* < 0.001) between Washington, NC and Orange, VA with both species being more commonly detected in Washington, NC. Cat weight was not significantly associated with *Bartonella* spp.: the mean weight of *Bartonella* spp.-positive cats was 2.93 kg compared to a mean weight of 3.26 kg for negative cats (*p* = 0.16). 

When utilizing a logistic regression model to analyze independent associations of location, weight, and sex with *Bartonella* spp. qPCR results, geographic location was the only variable independently associated with *Bartonella* spp.-positive PCR: cats in Washington, NC had 21.77 times higher odds of having one or more tissue *Bartonella* spp. qPCR positive (95% CI 6.65–86.45, *p* < 0.0001) compared to cats in VA. When utilizing the logistic regression model to analyze the associations of these same three variables (location, sex, and weight) with *B. henselae* or *B. clarridgeiae* amplification specifically, similar results were found with only location being significantly associated with detection of either species. 

### 2.3. Hemotropic Mycoplasma spp. 

**Figure 5:**
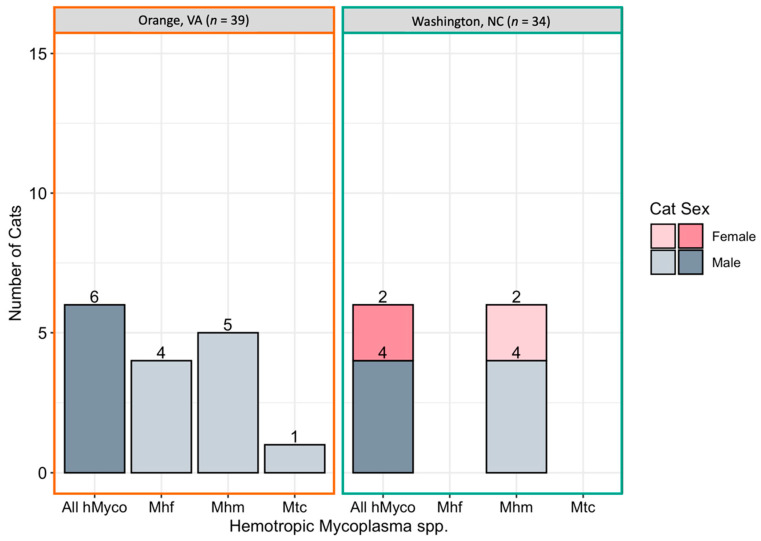
Number of cats infected with hemotropic *Mycoplasma* spp. by location. Cats were considered positive if one or more tissue samples were qPCR positive for hemotropic *Mycoplasma* spp. (All hMyco), *M. haemofelis* (Mhf), *M. haemominutum* (Mhm), and *Candidatus* M. turicensis (Mtc).

Overall, hemotropic *Mycoplasma* spp. (hMyco) DNA was qPCR amplified from one or more tissues from 12 of 73 cats (16%). Mhm was amplified from 11 cats, Mhf was amplified from four cats, and Mtc from one cat (Figure 5). Based on qPCR, two cats were coinfected with Mhm and Mhf and one cat was coinfected with Mhm, Mhf, and Mtc; all cats coinfected with multiple hMyco species were males from Orange, VA. The proportions of ear tip tissue and reproductive tissue positive for hMyco DNA on qPCR were equal, at 14% (10/73) (*p* = 1; Figure 3B). The type of tissues that tested positive for hMyco infection is summarized in Figure 2C,D for male and female cats, respectively.

Mhf DNA was amplified from ear tip and testicle tissues. Mhm DNA was amplified from ear tip, ovary, and testicle tissue. Mtc DNA was amplified only from testicle tissue. 

hMyco DNA was more likely to be amplified from male cats (10/37, 27%) than female cats (2/36, 6%; *p* = 0.02; Figure 3B). The proportion of cats with hMyco-positive qPCR was similar at both geographic locations, with 15% (6/39) of cats from Orange, VA hMyco PCR positive and 18% (6/34) of cats from Washington, NC (*p* = 1) hMyco PCR positive. The number of cats positive for hMyco by location and sex is summarized in Figure 5. Cat weight was significantly associated with hMyco infection: the mean weight of hMyco-positive cats was 4.31 kg compared to a mean weight of 2.90 kg for negative cats (*p* < 0.01). When only considering male cats, hMyco-qPCR-positive males (mean 4.71 kg) were heavier than the hMyco-negative males (mean 3.10 kg; *p* < 0.001). The two female cats were 2.65 kg and 2.05 kg, below the mean female cat weight of 2.72 kg (range 1.52–4.28 kg).

When utilizing the logistic regression model to analyze independent associations of location, weight, and sex with hMyco qPCR results, weight was the only variable independently associated with hMyco-positive PCR: for every 1 kg increase in weight, cats had 3.38 higher odds of being hMyco positive (95% CI 1.61–8.73, *p* = 0.00393). When utilizing the logistic regression model to analyze the associations of these same three variables (location, sex, and weight) with Mhm or Mhf amplification specifically, similar results were found with only weight being independently associated with the detection of either hMyco species.

### 2.4. Bartonella and Hemotropic Mycoplasma Comparison

When comparing hMyco to *Bartonella* spp., a significantly higher proportion of cats had *Bartonella* spp.-positive reproductive tissues than hMyco-positive reproductive tissues (35% vs. 13%, respectively; *p* < 0.01). In contrast, there was no statistically significant difference in the proportion of cats with ear tip tissue positive for *Bartonella* spp. versus hMyco (21% vs. 14%, respectively; *p =* 0.38). 

### 2.5. Inter-Genus Coinfection 

Coinfection of *Bartonella* spp. and hMyco was only documented in five cats. Mhm was the only hMyco found as a coinfection with a *Bartonella* spp. One male cat was coinfected with *B. henselae* (testicle) and Mhm (ear tip and testicle) and two male cats were coinfected with *B. clarridgeiae* (ear tip; ear tip and testicle) and Mhm (ear tip and testicle; ear tip). Both female cats who were infected with hMyco were also infected with a *Bartonella* spp.: one was coinfected with *B. koehlerae* (ovary) and Mhm (ovary), and the other was coinfected with *B. henselae* (ovary and uterus) and Mhm (ear tip). 

### 2.6. Fetal and Placental Samples 

Samples from fetuses and placentae were collected from 10 and nine queens, respectively. qPCR results from these tissues are provided in Table 1. Of the 20 fetal samples tested, 20% (4/20) were *B. henselae* qPCR positive. Of the 19 placental samples tested, 11% (2/19) were *B. henselae* qPCR positive. *Bartonella henselae* was the only *Bartonella* spp. amplified from fetal or placental samples.

## 3. Discussion 

In this study, we tested one ear tip and multiple reproductive tissues of free-roaming cats for DNA of multiple common feline vector-borne diseases. A significantly higher proportion of cats infected with *B. henselae* had *B. henselae* DNA amplified from reproductive tissue (19%) compared to ear tip tissue (5%, *p* = 0.02), whereas the same proportion of hMyco infected cats had hMyco DNA PCR amplified from ear tip (14%) and reproductive tissue (14%). In addition, only *B. henselae* DNA, not *B. clarridgeiae, B. koehlerae,* or hMyco DNA, was qPCR amplified from fetal or placenta tissues of pregnant queens. *Anaplasma, Ehrlichia*, and *Rickettsia* spp. DNA were not PCR amplified from any of the tissues tested from any cat.

It is important to consider whether the higher proportion of *B. henselae* PCR-positive reproductive tissues compared to ear tip may be influenced by the greater number of reproductive tissue samples tested, with an average of 2.75 reproductive tissue samples compared to a single ear tip sample per cat. However, despite the higher number of reproductive tissues tested, neither hMyco nor *B. clarridgeiae* displayed a greater rate of detection in reproductive tissues, indicating that the increased detection of *B. henselae* in reproductive tissues could be due to a greater pathogen burden in the blood or tissue tropism, and not simply greater sensitivity with testing of multiple samples. 

Despite limited literature investigating the tissue tropism of *Bartonella* spp., the ear tip appears to be a prime site for *Bartonella* spp. detection, and the skin has been identified as an important niche for *B. henselae* [42]. The ear tip has specifically been reported as a site of *Bartonella* spp. cutaneous disease manifestation, as shown by a recent case report of *B. henselae* associated with ear tip vasculitis in a dog [43]. Therefore, even equivalent detection in the reproductive tissue could be significant, as it represents a bacterial burden equivalent to one of the pathogen’s reported primary niches [44]. The higher proportion of *B. henselae* in reproductive tissues compared to ear tip tissue highlights the importance of focusing future studies on the reproductive system as a site of *Bartonella* spp. pathology. Despite the rising popularity of pet cats and cat breeding, infertility in domestic cats remains understudied compared to dogs and other domestic animals [45,46]. Given the cat’s role as the reservoir for multiple *Bartonella* spp. and previous studies suggesting *Bartonella* spp. as a cause of reproductive failure in cats [39], further investigation of the link between *Bartonella* spp., the feline reproductive tract, and infertility is warranted.

The detection of *Bartonella* spp. in the reproductive tract, fetus, and placenta of cats also has implications for the maintenance of *Bartonella* spp. in their cat reservoir host. Two studies have indicated a lack of transplacental transmission of *B. henselae* naïve queens inoculated with cultured *B. henselae* strains [39,47]. By inoculation of *B. henselae* just prior to or during pregnancy, these studies fail to mimic pregnancy in a reservoir host which is chronically infected or has experienced multiple reinfection events via chronic parasitism by *C. felis*. Abbott et al. documented seroconversion of kittens born to a bacteremic queen, presumably associated with transplacental transfer of immunoglobulins [47]. Kittens with maternal immunity were subsequently infected by inoculation with the same culture-grown strain that infected the queen, developing low bacteremia, or with a different *B. henselae* strain, developing high bacteremia [48]. Importantly, studies investigating reproductive outcomes following *Bartonella* spp. inoculation in cats have utilized culture grown *Bartonella* spp., which previous literature has indicated may result in reduced virulence [47,49,50,51]. Therefore, future work must carefully consider the inoculum source and timepoint(s) to ensure study outcomes that most closely mimic natural infection. 

Considering the human health implications of these findings, inoculation of rodents prior to pregnancy, whose discoid hemochorial placenta is more similar to that of humans than cats, with *B. birtlesii* indicated transplacental transmission was possible [40]. The sole case report supporting in utero or perinatal transmission of *B. henselae* in humans involved twins, one of whom died of a congenital heart defect (hypoplastic left heart syndrome) at 9 days of age [41]. Therefore, the present findings and previous work suggest future research should address if those *Bartonella* spp. associated with cats are able to be passed vertically from persistently bacteremic queens, as well as investigate the possible implications for *Bartonella* spp. as a pathogen of importance to human reproduction.

Hemotropic *Mycoplasma* spp. detection rates were identical by tissue type (ear tip and reproductive tissue for both male and female cats) for Mhm and Mhf, with Mtc being detected only in a testicle sample of a single cat, suggesting that these species do not display a predilection for either reproductive or ear tip tissue with equivalent detection by qPCR in both tissue types. This is expected as hMyco are erythrocytic organisms and both the skin and reproductive tissues are highly vascularized. Previous work has compared Mhf copy number in blood to those in specific tissue, determining that splenic and lung tissue harbored more Mhf; however, this work did not include reproductive or skin tissues [52]. Regarding host sex difference, the significantly greater detection of hMyco in cats of the male sex was expected, as it is an established pattern documented in several previous studies [19,53,54]. Failure of the logistic regression model to identify this pattern is likely due to the confounding relationship between cat sex and weight, as male cats (mean 3.53 kg) were significantly heavier than female cats (mean 2.72 kg). 

The detection of multiple hMyco in a single cat has also been previously reported, including triple co-infection with Mhm, Mhf, and Mtc as reported by Skyes et al. [19]. From a One Health perspective, sick humans are rarely tested for the presence of hMyco infections; however, there are several recent reports describing DNA of feline, canine, ovine, and swine-adapted hMyco in human patients [20,55,56,57]. Although a majority of hMyco infections are thought to cause minimal pathology in most infected species, it is of interest that most reported human cases have involved co-infection with *B. henselae* [20,55,58] while inter-genus coinfection was common in our cats. hMyco coinfection with *Bartonella* spp. in two female cats reinforces the importance and need for further investigation into the effects of coinfection on transmission and disease manifestation.

When interpreting the significant difference in the prevalence of *Bartonella* spp. between the two geographic locations from which cats were tested in this study, two factors must be considered: cat sex and true geographic differences in pathogen total and species-specific prevalence. While the proportion of male and female cats from Orange, VA and Washington, NC was not significantly different (*p* = 0.16), there was a slightly higher proportion of female cats sampled in NC compared to VA (58% vs. 41%). When stratified by location, female cats (85%) and male cats (36%) from Washington, NC had the highest proportion of *Bartonella* spp. infection, followed by female (19%) and male (6%) cats from Orange, VA. Female cats from the same location as male cats consistently displayed higher infection prevalence, indicating a confounding relationship between sex, location, and *Bartonella* spp. infection in this study. Previous literature has not suggested a difference in *Bartonella* spp. infection prevalence between male and female cats [59,60], indicating that our sampling methodology may have resulted in increased detection of infection in females. This could reflect sampling bias, since more tissues per cat were tested for females than males (mean 4.5 tissues samples for females vs. two tissue samples for males) or due to a true difference in the pathogen’s presence in tissue, which would not be reflected in previous literature, which commonly utilizes blood samples for diagnostic assays. 

The exclusive detection of Mhf and Mtc in Orange, VA, despite the similar rate of Mhm detection between locations, indicates that this observation may be attributed to true difference in prevalence between locations. The unexpected differences in the prevalence of both *Bartonella* spp. and hMyco between these relatively close (322 km) locations indicates that these species may display precise geographic variations in prevalence. This would be an important consideration when locations are grouped together in regional or national prevalence surveys. 

The limitations of the current study included the sampling of only two locations, sampling at different times of the year, lack of accurate cat age data, and sampling by two different veterinary teams. All of these factors may have introduced variation in pathogen prevalence between locations. By only sampling two locations, we are unable to draw conclusions about the broader geographical patterns of *Bartonella* and hMyco species among cats in other regions. Sampling Washington, NC in March and Orange, VA throughout the year may have confounded our results if these species display varying prevalence throughout the year. The sampling of an unequal number of reproductive tissues (average 2.75 per cat) versus ear tip tissues (one per cat) may have resulted in an increased detection in reproductive tissues. While our microbiological results are specific based upon sequencing of amplicon DNA, a lack of sensitivity with qPCR likely resulted in some infected cats being considered uninfected [61]. 

## 4. Methods 

### 4.1. Study Design and Sample Sources

The present study was performed as a prospective cross-sectional observational study of FRC ear tip and reproductive tissues collected from cats located in Washington, North Carolina in Beaufort County and Orange County, Virginia. Trap-neuter-release (TNR) programs were already in place to control the FRC populations locally and improve FRC welfare [62]. Volunteer veterinary personnel collected tissues removed for spay/neuter while cats were anesthetized. Samples from Washington, NC were collected in collaboration with Paws and Love, Inc. in March 2019. Samples from Orange, VA were collected in collaboration with the Orange County Humane Society TNR program and Paradocs Animal Hospital in May 2019 and November 2020 through January 2021. Ear tip biopsy was performed for the purpose of identifying FRCs that had previously undergone spay or neuter [62]. This study was approved by the North Carolina State University IACUC (Protocol #19–003). 

### 4.2. Data and Specimen Collection 

Cats were selected for study participation upon presentation to TNR program regardless of sex or ectoparasite infestation. All cats anesthetized for spay/neuter during the study time periods were enrolled. For the primary objective, cats were excluded if ear tip tissue was not collected, or if less than two testicle (male) or two ovary (female) samples were collected. Therefore, all cats had at least one ear tip and two reproductive tract tissue samples available for testing. For the secondary objective, only pregnant queens with at least 1 sample from the queen reproductive tract and 1 fetus sample were included. Demographic data including sex and weight were recorded by veterinary personnel at each location. 

Following routine spay or neuter surgical procedures and ear tip removal by the attending veterinarian, the reproductive tracts (left and right ovary and uterine horn for females, left and right testicle for males) and ear tips were collected and labeled. All reproductive tissues from an individual cat were stored in the same container for transport, and each ear tip was stored individually. Shortly after collection, tissues were frozen and transported to the Intracellular Pathogens Research Laboratory (IPRL) in a Styrofoam box with ice packs. Samples remained frozen (−20 °C) until dissected for DNA extraction. Dissection was performed utilizing a disposable scalpel and tweezers, which were sanitized with 100% ethanol between samples in order to reduce the possibility of DNA carryover between tissues [63]. The tissues dissected from each organ included: one 1 × 1 cm section from the center of each ovary, one 1 × 1 cm section from each uterine horn, one 1 × 1 cm section from the center of each testicle, and a 1 × 1 cm section of the ear tip. Due to loss of samples during collection, uterus tissue was not available for 9 cats from NC; all other cats had all tissues sampled. Figure 1 provides a summary of the tissues collected from each location. 

For investigation of *Bartonella* spp. in fetal and placental tissue, cats from whom fetal and/or placental tissues were collected were reported regardless of ear tip sample availability. For those early in gestation, the entire fetus was utilized for DNA extraction, while an approximately 1 × 1 cm sample from across the mid-abdomen was selected for later gestation fetuses. Fetal and placenta tissues underwent the same processing and qPCR procedures detailed below. 

DNA extraction from tissues was performed utilizing the Qiagen DNeasy Blood and Tissue Kit (Qiagen, Valencia, CA, USA) following manufacturer’s tissue extraction protocol. DNA concentration (ng/μL) and purity (A_260_/A_280_) were determined spectrophotometrically (Thermo Fisher Scientific, Waltham, MA, USA). 

### 4.3. Pathogen Detection

A housekeeping qPCR amplifying the glyceraldehyde-3-phosphate dehydrogenase (GAPDH) gene was performed to ensure the presence of amplifiable DNA and to assess for potential PCR inhibition [64]. All samples had amplifiable DNA by the housekeeping qPCR. 

Pathogen detection was performed with PCR reactions amplifying the *Bartonella* spp. *ssrA* gene [65] and 16S–23S intergenic spacer (ITS) region [61,66,67], hemotropic *Mycoplasma* spp. 16S rRNA region (this study), *Rickettsia* spp. 16S-23S ITS region [65], and *Anaplasma* and *Ehrlichia* spp.16S rRNA gene [68]. qPCR conditions for the detection of hemotropic *Mycoplasma* spp. are detailed below, while other assays were performed as detailed in cited literature. Primer sequences are shown in Table 2. 

Hemotropic *Mycoplasma* spp. amplification reaction was performed in a 25 µL reaction consisting of 12.5 µL of SYBR Green Supermix (Bio-Rad), 5 µL of DNA, 0.1 µL of each primer at 50 µM, and 7.3 µL of molecular grade water. The reaction began with 3 min of denaturation at 98 ℃, then 35 cycles of 98 ℃ for 15 s, 62 ℃ for 15 s, and 72 ℃ for 15 s, and finally, a melt curve analysis from 65 ℃ to 90 ℃ at 0.5 ℃ increments. Validation confirmed detection of Mhf, Mhm, Mtc, *Mycoplasma haematoparvum, Mycoplasma haemocanis,* as well as additional hemotropic *Mycoplasma* spp. found in rhinoceros, antelope, llama, and bovine. The primers did not detect bacteria belonging to *Anaplasma, Bartonella, Ehrlichia,* or *Rickettsia* spp. 

Three controls were included in each reaction, a non-template control consisting of molecular grade water, a negative control from a cat known to be negative for all PCR targets, and a positive plasmid control or bacterial culture control. Reactions were performed in a CFX96 Touch Real-Time Detection System (Bio-Rad, Hercules, CA, USA). Sequencing of all positive samples was performed at GENEWIZ Inc. (Raleigh, NC, USA), analysis and editing of chromatograms performed utilizing SnapGene (Insightful Science, San Diego, CA, USA), and NCBI nucleotide BLAST (National Center for Biotechnology Information, Bethesda, Maryland, USA) was utilized to compare sequences to known species. 

A cat was considered PCR positive for a given pathogen if ≥1 of the cat’s samples generated an amplicon that could be confirmed to have >99% similarity by DNA sequencing. For *Bartonella* spp., a cat was considered positive if either the *ssrA* or ITS gene target was successfully amplified. 

### 4.4. Statistical Methods

Descriptive statistics (mean and standard deviation for continuous data and proportions) were calculated. The proportion of PCR positive cats for each pathogen was compared by geographic location, tissue type, sex, and cat weight to determine associations. Fisher’s exact test was employed when examining categorical variables and two sample t-test employed for normally distributed continuous variables. Two multivariable logistic regression models examining the likelihood of a cat having one or more tissue samples positive for (1) *Bartonella* spp. or (2) hemotropic *Mycoplasma* spp., as the dependent variables were used to calculate adjusted ORs for explanatory variables (geographic location, sex, and weight). Values of *p* < 0.05 were considered statistically significant. All analysis was performed in R, version 4.0.4 (R Foundation for Statistical Computing, Vienna, Austria) in Windows utilizing the packages cowplot [69], ggthemes [70], here [71], irr [72], janitor [73], stringr [74], and tidyverse [75,76].

## 5. Conclusions

This study provides evidence that *B. henselae* is more common in the reproductive tissue than the ear tip tissue of cats. This finding combined with *B. henselae* amplification from the fetal and placental samples of pregnant queens highlights the need for further investigation into the role of vertical transmission in the maintenance of *B. henselae* in this reservoir host, as well as any possible effects of *Bartonella* spp. infection on the reproduction of both cats and other mammalian species. Significant differences in *Bartonella* spp., Mhf, and Mtc detection between two sites in the South Atlantic United States indicates there may be precise geographic patterns of pathogen prevalence, which remain to be elucidated. Future research should focus on detailed pathologic and bacterial imaging analysis of infected reproductive and fetal tissues, attempted isolation of viable bacteria from fetal tissue, and in vivo studies of queens known to be infected with *Bartonella,* hemotropic *Mycoplasma,* or members of both genera.

## Figures and Tables

**Table 1:** *Bartonella* spp. qPCR results from cats with fetal or placenta tissue collected. Total number of positive samples divided by the total number of samples collected is reported in the second column. N/A, tissue not sampled for the cat(s). Neg, tissue was tested and was negative.

Number of Cats	Positive/Tested	Ovary	Uterus	Fetus	Placenta	Ear Tip
1	3/8	*B. henselae*	Neg	*B. henselae*	*B. henselae*	Neg
1	2/8	*B. henselae*	Neg	Neg	*B. henselae*	N/A
1	2/10	*B. henselae*	Neg	Neg	Neg	Neg
1	1/7	*B. henselae*	Neg	N/A	Neg	Neg
1	1/7	Neg	*B. clarridgeiae*	Neg	Neg	Neg
1	2/8	Neg	*B. henselae*	*B. henselae*	Neg	N/A
1	1/7	Neg	Neg	*B. henselae*	N/A	Neg
1	1/8	Neg	Neg	*B. henselae*	Neg	N/A
2	0/8	Neg	Neg	Neg	Neg	N/A
1	0/7	Neg	Neg	Neg	N/A	Neg

**Table 2:** Primers targeting housekeeping genes and pathogens. GADPH: glyceraldehyde-3-phosphate dehydrogenase, ITS; intergenic spacer, FAM: 6-fluorescein amidite, IABkFQ: Iowa Black ^®^ FQ.

Target Organism	Oligonucleotide Name	Oligonucleotide Sequence (5′-3′)	Target Gene	PCR Product Size (bp)	Reference
Housekeeping	HKA_GAPDH	CCTTCATTGACCTCAACTACAT	GADPH	357	[64]
	HKB_GAPDH	CCAAAGTTGTCATGGATGACC			
*Bartonella* spp.	Bart_ssrA_F	GCTATGGTAATAAATGGACAATGAAATAA	*ssrA*	158	[65]
	Bart_ssrA_R3	GACAACTATGCGGAAGCACGTC			
*Bartonella* spp.	BsppITS325s	CCTCAGATGATGATCCCAAGCCTTCTGGCG	ITS	130	[61,66,67]
	BsppITS543as	AATTGGTGGGCCTGGGAGGACTTG			
	BsppITS500p	FAM-GTTAGAGCGCGCGCTTGATAAG- IABkFQ			
*Rickettsia* spp.	Rick23-5_F2	AGCTCGATTGATTTACTTTGCTG	23S-5S	247	[65]
	Rick23-5_R	TTTGTATTGCTAGCTTGGTGG			
*Mycoplasma* spp.	Myco_Hf_F.1	GACGAAAGTCTGATGGAGCAAT	16S rRNA	127	This study
	Myco_Hf_R	ACGCCCAATAAATCCGRATAAT			
*Anaplasma* and *Ehrlichia* spp.	AE16S_45F	AGCYTAACACATGCAAGTCGAACG	16S rRNA	199	[68]
	AE16S_299R	CCTCTCAGACCAGCTATAGATCA			

## Data Availability

Data is available via Dryad (https://doi.org/10.5061/dryad.x69p8czk4).
